# Affinity Ionic Liquids for Chemoselective Gas Sensing

**DOI:** 10.3390/molecules23092380

**Published:** 2018-09-18

**Authors:** Albert Chang, Hsin-Yi Li, I-Nan Chang, Yen-Ho Chu

**Affiliations:** 1 Department of Chemistry and Biochemistry, National Chung Cheng University, 168 University Road, Minghsiung, Chiayi 62102, Taiwan; changalbertalbertchang@gmail.com (A.C.); lanbarla0708@gmail.com (H.-Y.L.); 2 ANT Technology Co., Ltd., 137, Section 1, Fushing South Road, Taipei 10666, Taiwan; ant.tech@msa.hinet.net

**Keywords:** volatile organic compound, chemoselective gas analysis, ionic liquid, quartz crystal microbalance

## Abstract

Selective gas sensing is of great importance for applications in health, safety, military, industry and environment. Many man-made and naturally occurring volatile organic compounds (VOCs) can harmfully affect human health or cause impairment to the environment. Gas analysis based on different principles has been developed to convert gaseous analytes into readable output signals. However, gas sensors such as metal-oxide semiconductors suffer from high operating temperatures that are impractical and therefore have limited its applications. The cost-effective quartz crystal microbalance (QCM) device represents an excellent platform if sensitive, selective and versatile sensing materials were available. Recent advances in affinity ionic liquids (AILs) have led them to incorporation with QCM to be highly sensitive for real-time detection of target gases at ambient temperature. The tailorable functional groups in AIL structures allow for chemoselective reaction with target analytes for single digit parts-per-billion detection on mass-sensitive QCM. This structural diversity makes AILs promising for the creation of a library of chemical sensor arrays that could be designed to efficiently detect gas mixtures simultaneously as a potential electronic in future. This review first provides brief introduction to some conventional gas sensing technologies and then delivers the latest results on our development of chemoselective AIL-on-QCM methods.

## 1. Introduction

Gas, one of the four fundamental states of matter, has a unique place in the eye of human. To say the least, the air human inhale contains oxygen that serves as an oxidizing agent in cellular respiration to release adenosine triphosphate (ATP), the molecular unit of currency for our everyday activity. Also, the carbon dioxide exhaled by human is the main ingredient for plants to undergo photosynthesis and release oxygen back to the atmosphere. Analysis of gases is of great importance for a myriad of applications in health, safety, military, industry and environment. For example, breath volatile organic compounds (VOCs) have found immense value in noninvasive disease diagnosis and metabolism monitoring [1,2,3,4,5,6,7,8]. Food industry uses gas sensing technologies for quality control of fruit ripeness and detection of meat spoilage [9,10,11,12,13]. Analysis of indoor air quality domestically and industrially has been implemented for monitoring hazardous and asphyxiant gases [14,15,16]. Moreover, selective detection of gases is used as means of both homeland securities for early detection of chemical warfare agents (CWAs) [17] and industrial regulation for greenhouse emission [18]. As a result, it is obvious that, in sync with the advance of science technology, increase in human activities, improvement in life quality and health, demands for gas analysis will only continue to grow. However, to provide quantification analysis of gases is rather difficult than it may sound.

Unlike liquid or solid, gas is shapeless and invisible to the naked human eye due to the large discretion between each molecule, allowing them to move quickly and freely. To add on, the abundance of gaseous molecules such as single atom gases (e.g., noble gases: He, Ni and Ar), single type of atom gases (e.g., O_2_ and N_2_) or multiple atom gaseous molecules (e.g., CH_4_ and CO_2_) augments the difficulty for specific trace detection. Therefore, considering the complexity of the constituents in gas mixtures along with other properties, the importance to develop capable gas sensing methods has never been more on demand.

**Figure 1:**
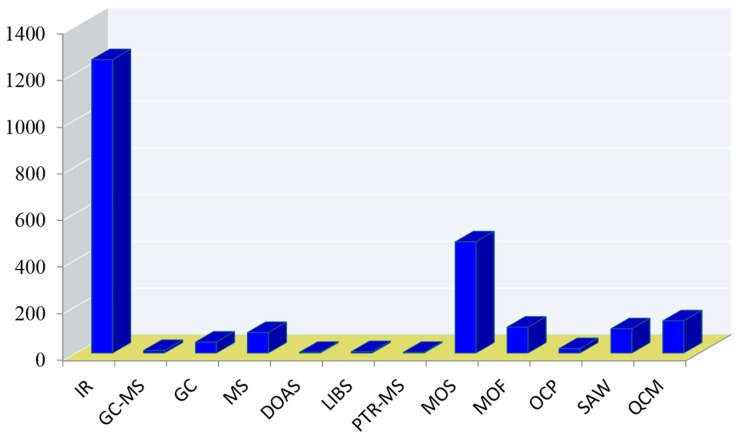
Number of articles published in English on the subject of analytical tools used for “gas sensing,” as determined by SciFinder on 18 August 2018. IR, infrared spectroscopy; GC-MS, gas chromatography-mass spectrometry; DOAS, differential optical absorption spectroscopy; LIBS, laser-induced breakdown spectroscopy; PTR-MS, proton-transfer reaction mass spectrometry; MOS, metal oxide semiconductor; MOF, metal organic framework; OCP, organic conducting polymer; SAW, surface acoustic wave; QCM, quartz crystal microbalance.

Gas analysis has long been an important field of study that can be dated back to Hippocrates for his study on breath aroma [19]. Yet, due to the lack of sufficient detection methods and tools, the development of gas analysis had largely stand pat at qualitative analysis. With the advances in science and technology, scientists had started to employ new devices capable of providing quantitative analysis. Most notably, in 1950s, Keeling and coworkers deployed an infrared gas analyzer to give birth to the renowned graph, the Keeling Curve, which was first to provide the direct linkage of human activities to climate change in continuous measurement of carbon dioxide concentration for 20 years [20]. In 1971, Pauling and coworkers spent 3 years to develop a gas-liquid chromatography system that permits the determination of more than 250 substance in breath and urine vapor, signaling the start of modern breath analysis [21]. Since then, great strides have been made in the development of analytical methods, many of which are powerful tools that provide comprehensive information on gas analysis. A brief survey of the available tools is provided in Figure 1. However, given the wide ranges available, not one is without flaw and can be satisfied for all needs. In this review, general description on common gas sensing methods is provided, along with ionic liquids (ILs), the materials that have garnered our interest due to their unique properties. Finally, a detail account is reported on affinity ionic liquid (AIL) by quartz crystal microbalance (QCM), a chemoselective sensing method with great potential for commercial use.

**Figure 2:**
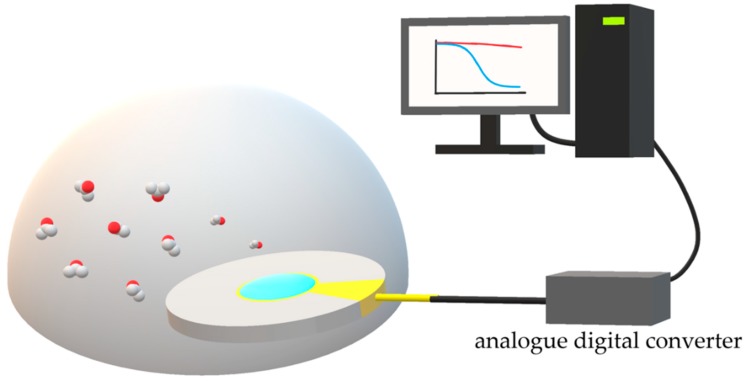
Schematic representation of an AIL-on-QCM gas analysis system.

A complete sensing scheme may be divided into five parts. First, an inert reference gas such as N_2_ for baseline determination serves as a sample carrier. Second, a chamber is used for introduction of sample via injection or headspace sampling. Third, physical or chemical adsorption of gaseous sample on sensing substrate will result in either a physical, optical, or electrical change. Then, an analogue digital converter capable of reading the physical, optical, or electrical change will convert it into digital signals for computer analysis. Last, data analysis is proceeded with mathematical algorithm. A basic flow diagram is depicted in Figure 2. In this review, the bulk sensing methods are categorized into four domains: spectroscopic, spectrometric, conductivity and piezoelectricity methods. It is also worth noting that these techniques can be used together via an interdisciplinary approach. In addition, the pros and cons of the techniques will be provided for the evaluation of its selectivity, sensitivity, cost, response time and potential for real-time application.

## 2. Gas Sensing Methods

### 2.1. Spectroscopic Methods

There are many spectroscopic methods available in the sensing of gas molecules, including differential optical absorption spectroscopy (DOAS) and Fourier transform infrared spectroscopy (FT-IR), many of which that have been applied in field test [22,23]. The principle behind most spectroscopic gas analysis is based upon either absorption spectroscopy or emission spectroscopy. Absorption spectroscopy can be comprehended via the Beer-Lambert’s law, in which the analytes can be characterized by the wavelength or frequency when absorption occurs. DOAS is an example of this and is widely used in atmospheric science for its low limit detection and good accuracy. However, DOAS suffers from interference such as NO_2_ and stray lights or unremoved sets in the spectra [24]. Emission spectroscopy involves the emission of photons when a molecule is excited and then returned to ground state and such an example is the laser-induced breakdown spectroscopy (LIBS). In short, spectroscopic methods have the advantage of being able to provide high selectivity and sensitivity detection. They however suffer from large instrumentation and high cost. Additional development of miniaturized instruments with affordable cost will be the next step forward.

### 2.2. Spectrometric Methods

Another conventional method for gas analysis is the gas chromatography-mass spectrometry (GC-MS). Evidence through its namesake, GC-MS is composed of two instruments: a gas chromatography system that is responsible for separation of the molecules in gaseous sample and a mass spectrometer that provides analysis of the molecules through fragmentation of the molecules by ionization to obtain mass spectra which can be then correlate to a fingerprint in the database for determination. Yet, given its advantages against other methods, the GC-MS is handicapped largely by many factors. These include the lengthy pre-concentration and analysis duration needed that make GC-MS inapplicable for real-time performance. Also, constant calibration needed by a knowledgeable operator makes it difficult to popularize [25]. Nevertheless, developments have been made in hope of refining the methods.

The solid-phase micro extraction (SPME) is a sample pre-treatment technique developed to provide a simple, fast and solvent free preparation of the analytes to cut down the lengthy pre-concentration process [26]. A highly selective polymer coated fused-silica fiber is immersed into liquid or by exposure in the headspace for desired molecules to be adsorbed onto. This allow elimination of impedance by solvent peaks and has thus been used in many fields. Further modification of the fiber is also available to improve selectivity of the sample [27,28]. Albeit it was successful in decreasing the time needed, it is still not appropriate enough for real-time application [29,30].

Selected ion-flow tube (SIFT) is another method developed to provide better real-time analysis through alternative ionization method. It requires the chemical ionization of trace gas in air samples. The samples mixed with thermalizing buffer gas are then carried through for detection by the mass spectrometer. The method provides real-time, high sensitivity analysis of the molecules, yet, it fails to detect compounds of the same m/z ratio. Nevertheless, it is still widely applied in fields, including an on-line quantification of VOCs at the headspace of roasted coffee by Dryahina and coworkers [31], air quality determination at drug storage areas by Doran and coworkers [29], and breath analysis by Spanel and Smith [32].

Proton-transfer reaction mass spectrometry (PTR-MS) is developed for better detection of gaseous organic compounds. It relies on the chemical ionization of gas sample by proton transfer (H_3_O^+^) inside the drift tube. Furthermore, a more compact instrumentation relative to SIFT with the advantages of higher sensitivity, online measurement with no requirements of pre-concentration and substance calibration is provided. Due to its proton-transfer thermodynamics, PTR-MS receives no interference with photon affinities [33]. However, this also implies that small molecules such as CO, CO_2_ and methane cannot be detected using standard PTR-MS [34]. Although it provides superior size advantage and user-friendliness in comparison to other MS methods, it is still considered bulky. In addition, similar to SIFT it also fails to differentiate isobaric compounds.

### 2.3. Conductivity Based Methods

#### 2.3.1. Metal Organic Semiconductors

Metal oxide semiconductors (MOSs) are solid-state gas detecting devices commonly used in industry for its low-cost, easy production and compact size [35,36]. The working principle of MOS is straightforward: the measurement of changes in conductivity of the metal oxide layer when interaction (e.g., redox reactions) with the surrounding environment occurred. Furthermore, high sensitivity by MOSs have led to numerous applications in gas sensing of nitrogen oxides, sulfur dioxide, hydrogen sulfide, hydrogen, ozone, VOCs and CWAs [37]. However, these sensors usually suffered from poor selectivity given their vulnerability to poisoning (e.g., ethanol, volatile sulfur compounds and humidity) [38]. In addition, high temperature (>400 °C) required for operation and the extensive functionalization needed to further improve sensitivity also hampers its practical applications. To tackle aforementioned problems, recent research works have demonstrated promising improvements in selectivity and thermal stability through the doping of nanostructures (e.g., nanotubes and nanowires) and nanocomposites (e.g., Pt, Nb, CeO_2_, or PdO) or by structure modification to increase surface area [39,40,41]. However, it has to be mentioned that besides the exact working mechanisms for modified MOSs via doping remain uncertain, there are still multiple aspects that needs to be addressed. In a recent review detailed by Korotcenkov and Cho, these aspects include: small to little improvements in gas sensing sensitivity, limited reproducibility due to the complex material involved, careful monitoring for large number of parameters needed for optimal control of the material and the trade-off of other parameter of sensor for improvement in selectivity and sensitivity [42]. Therefore, it is clear that further efforts need to be made in understanding the mechanism of the conductivity response occurred at the metal oxide layer.

#### 2.3.2. Metal Organic Framework

In part of their highly tunable and diverse structures, metal organic frameworks (MOFs) have garnered increasing interests over the past years. Furthermore, an outburst of development directed at numerous fields has been made, including catalysis, gas storage, separation, electric capacitors [43], energy storages [44], lithium ion batteries [45] and chemical sensors [46,47]. Given our focus in sensing tools, we will look into solely on its recent development as gas sensing methods.

As one can expect from its versatility to multiple fields, many facets of MOF properties have been experimented for possible gas sensing applications. For example, its low conductivity has led to the introduction of impedance spectroscopy in detecting different gases. Although recent success has been made in detecting ammonia and methanol, studies for better selectivity are still required for further applications [48,49,50,51]. Another application that utilized low conductivity of MOFs is its role as a chemiresistive sensor. Initial works in the field have displayed slow recovery time and high temperature requirement [52]. However, recent works by Campbell and coworkers have shed light through introducing new fabrication methods of the MOFs that were able to discriminate different VOC vapors [53]. Other works have also provided improvements through various fabrication methods [54]. Nonetheless, it should be noted that such method is still at its embryonic stage and the exact mechanisms are unknown and further studies are required [55].

Optical responses are another way MOFs can contribute in gas sensing. The method involves changes that are detected based upon either a shift in the emission spectrum, or a change in luminescence intensity. The former can be achieved by two passages: a change in solvent polarity and a change in the coordination environment of the metal ion. The latter usually involves the quenching (turn-off) or enhancement (turn-on) of photo-induced emission due to guest adsorption, with the effectiveness determined by the nature of the guest-host interactions [56]. The good sensitivity and regenerability have led to success in sensing numerous gaseous molecules, as reviewed in literature [57,58,59]. However further development is required for it to become fully applicable as its limitations include medium stability and the insufficient knowledge to fully address the selective nature of this method [46,60]. In addition, MOFs, as a selective sorbent layer, can be incorporated with mass-sensitive sensors, such as acoustic wave sensors, to achieve better selectivity of analytes.

#### 2.3.3. Organic Conducting Polymer

Other than the MOSs and MOFs, organic conducting polymer (OCP) is another conventional method that has been studied extensively [61,62]. However, due to low conductivity and stability of organic materials, further fabrication is required to enhance conductivity through protonation or redox reactions, thus lead to many nanocomposite derivatives [61]. A typical OCP sensor setup constitutes of two electrodes fabricated with an insulating polymer. Modulation of the insulating polymer via spraying, spinning, or coating allows improvement in conductivity. Once exposed to gas, physical properties of the insulating substrate changes due to gaseous molecule interaction with the sensing substrate upon adsorption, thus a change in resistance occurs and measurements can be made accordingly [62]. In comparison to its inorganic conducting counterparts, OCP sensors have the advantage of short response time at room temperature with high sensitivity. Moreover, the adoption of nanocomposites increases their surface-volume-ratio to decrease response time. Thus, an array of OCPs, acting as an e-nose system, can lead to commercial applications for real-time analysis such as continuous industrial hazardous VOCs monitoring [11]. However, the improvement in response time is upstaged by the low reproducibility, selectivity and stability of the OCP structure. Furthermore, the conductivity of polymers can also be influenced by many factors, including temperature fluctuation [63]. In addition, as mimics of enzyme-substrate complexes, molecularly imprinted polymers (MIPs) can provide high selectivity for adsorption of target analytes with template-tailored cavities. These structure-directed sensing materials have been reported to successfully analyze organic molecules for determination of optimal harvest maturity in fruits. Nonetheless, the complete removal of analytes from the MIPs can be challenging and limits its productivity [64].

### 2.4. Piezoelectric Methods

#### 2.4.1. Surface Acoustic Wave

Surface acoustic wave (SAW) sensors and bulk acoustic wave (BAW) sensors are two of the most used methods applying piezoelectric effect. SAW sensors differ from BAW sensors in that its acoustic wave mainly propagates and is confined within the near surface region, typically beneath one wavelength of the surface, whereas the wave of BAW sensors covers the whole body [65,66]. Nevertheless, the sensing mechanism for both is similar: the measurement and analysis of the change in wave frequency resulted from chemical or physical adsorption at the substrate.

**Figure 3:**
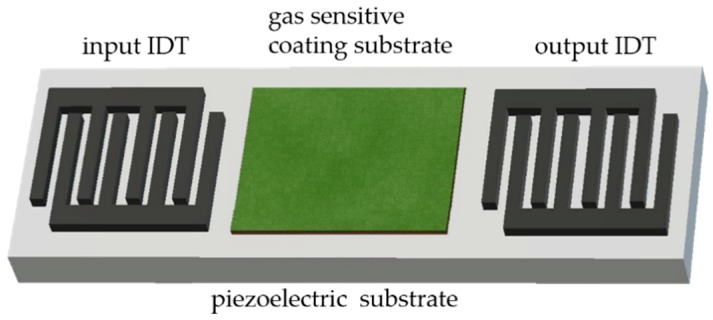
A brief configuration of SAW sensor. The coating substrate can be of polymer, MOS, MOF, nanocomposites and others.

The basic components of a SAW sensor include an input interdigitated transducer (input IDT), an output interdigitated transducer (output IDT) and a gas-sensitive coating substrate on piezoelectric substrate, as illustrated in Figure 3. An input IDT set at one end of the substrate initiates an acoustic wave that propagates along the surface of the substrate to the output IDT located on the other end. Midway through the propagation, a change of the coated region upon interactions of the gas molecules at the surface of the substrate will result in a change in the oscillation frequency. The output IDT receives the signal and outputs it to an analyzer for further computation. The interactions involved usually result to a change of mass, viscoelasticity and conductivity that will cause a time delay between the input and output IDTs, thus a wave shift that can be correlated proportionally with mass will occur, giving it its high sensitivity [67,68,69].

The modulation of the propagation path is crucial component of a SAW-based sensor. In fact, the selectivity, sensitivity and repeatability largely depend on the sensing materials deposited on the piezoelectric substrate. Common coating materials include polymers, MOSs, MOFs, carbon nanotubes and nanoparticles. Polymer films have shown to provide short response and recovery time through physical adsorption of gas molecules. However, poor selection requires a better structure and doping. Metal oxides and MOFs, given by their high thermal stability, can detect inorganic gases and VOCs through redox reactions at high temperatures [69]. Carbon-nanotubes provide high sensitivity at room temperature due to their large surface to volume ratio, providing quick response and high adsorption capacity.

#### 2.4.2. Bulk Acoustic Wave

Quartz crystal microbalance (QCM) belongs to the larger group of BAW that has been studied extensively and applied to many fields. For example, coated QCM with antibody and gold nanoparticles has been used recently for disease diagnosis and antigen detection in solutions [70,71,72,73,74]. Study of cells can be conducted through the examination of cell-substrate adhesion with QCM [75,76]. Furthermore, detection of environmental pollutants can also be done with QCM immunosensors [77]. The high sensitivity, stability, fast-response and low-cost characteristics have made QCM one of the most common tools applied.

**Figure 4:**
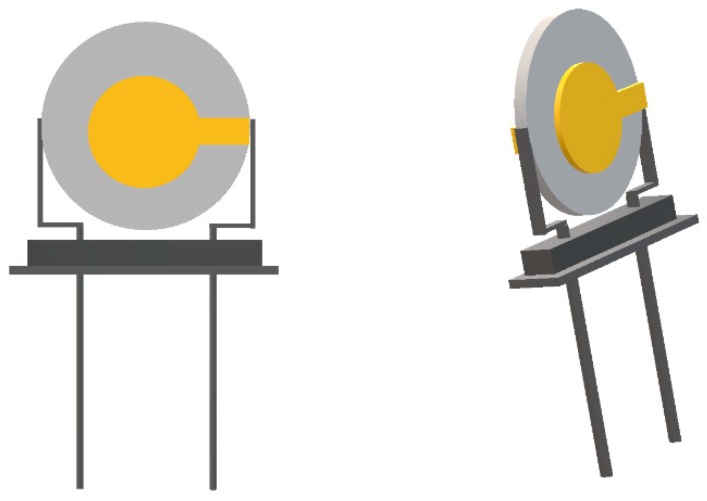
Schematic representation of a QCM sensor chip.

Unlike a ST-cut quartz used for SAW sensors, the QCM is made up of an AT-cut quartz sandwiched between two electrodes that is usually attached to an AC voltage, as shown in Figure 4. Upon molecular adsorption or reaction at the surface, a shift in the oscillating frequency of the quartz substrate occurs, indicative of a change in mass [78]. This working mechanism in gas phase is depicted by the Sauerbrey equation [79], where the shifting oscillation frequency can be correlated to the change of mass. Later, Kanazawa and Gordon derived a new equation for fluids based on the Sauerbrey equation, allowing further applications [80]. Similar to SAW, high sensitivity and yet low selectivity of the device also imply low tolerance to slight changes in the environment, making it highly susceptible to interference. Nevertheless, developments have been made for different uses of QCM. For example, to account for viscoelasticity common to biological molecules, inclusion of dissipation led to the introduction of QCM dissipation (QCM-D). Electrical QCM (EQCM) can be used to characterize electrochemical process. Moreover, carbon nanotubes, polymers, MOSs, MOFs and ionic liquids can also be fabricated, deposited on QCM sensors to improve performance.

QCM-D is an often-used technique developed to provide better analysis of biological molecules but hardly applied in gas sensing. The technique relied on the monitoring of dissipation to obtain information on the structure of the substrate. This is achieved by turning off the power of the oscillating crystal and allows the oscillation decay to be measured [81,82,83]. A combination of the dissipative and resonant frequencies allows better estimation of the viscoelastic mass.

EQCM utilizes both amperometric sensing and mass sensing to provide cross validation in analysis. For an electrochemical experiment, three electrodes are immersed in liquid phase to perform methods such as cyclic voltammetry, differential pulse voltammetry, or square wave voltammetry. Most applications of EQCM have been focused on fields related to characterization of biological molecules and energy storage studies [84,85,86,87]. Furthermore, the introduction of ionic liquids, which provide features such as conductivity and great adsorption ability for gases, has opened a new door for new applications with EQCM. Yu and coworkers were able to employ ionic liquids as both electrolytes and sorption solvents for cross analysis of ethyl nitrobenzene [88].

Better sensing through fabricating the surface of the substrate to achieve higher selectivity and sensitivity remains to be the most dominant pathway in gas sensing with QCM. Likewise, the incorporation of MOSs and MOFs allowed improved sensitivity and selectivity through redox reaction and high surface ratio but required higher temperature and longer response time. The incorporation of molecularly imprinted polymers on QCM also showed high affinity and sensitivity toward target molecules [89]. Polymers through their porous structures are another commonly applied method for better selectivity [9,90,91]. Nanostructured modified QCM was also used for successful detection of plasticizer vapors, hydrogen sulfide and ammonia [92,93,94].

Although surface modifications of QCM provide various advantages in gas sensing, it also brought along its disadvantages to be solved. How to provide further modifications for better sensitivity, selectivity and reproducibility under ambient conditions for real-time applications remains as a challenging task. Ionic Liquids, with its many advantages, have the potential to fill this need.

## 3. Ionic Liquids and Its Use for Adsorption Analysis of Gases on QCM

**Figure 5:**
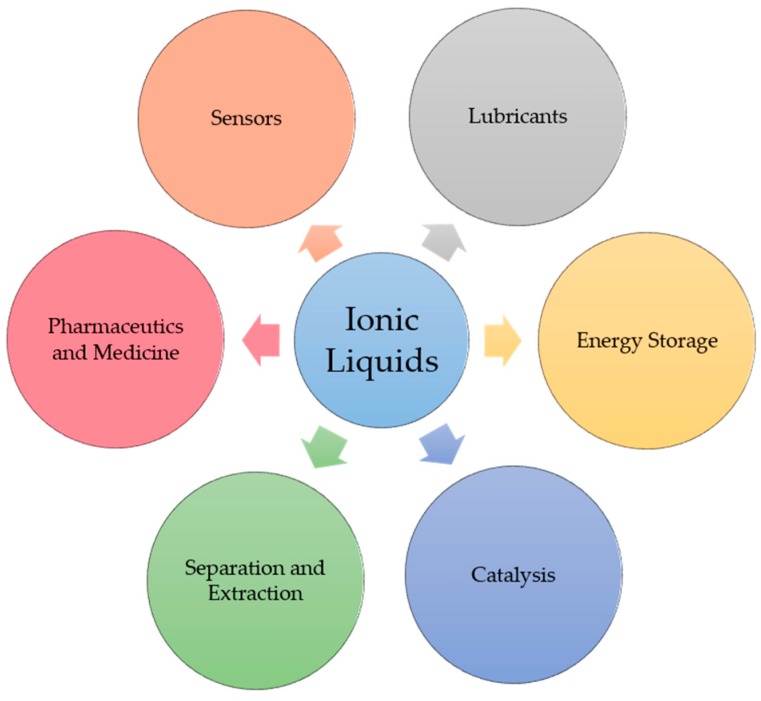
Common applications of ionic liquids.

**Figure 6:**
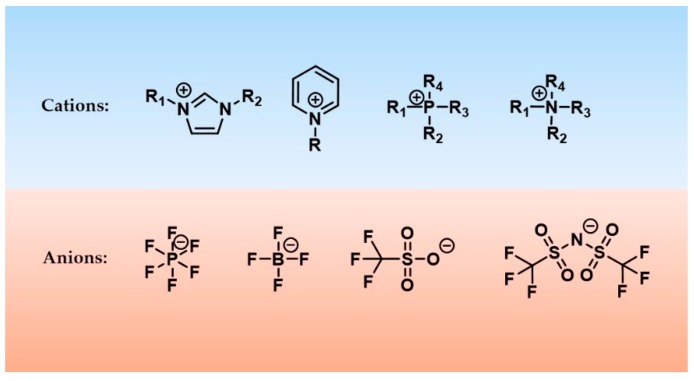
Common structures of ionic liquids.

Room temperature ionic liquids (RTILs) are liquidus molten salts at ambient temperature and composed entirely of ions. Their diverse properties in providing excellent physical, chemical, thermal and electrochemical stability, nonflammability, very low vapor pressure, good solubility and tunability have garnered them significant studies in wide range of fields (Figure 5) [95,96]. For example, tailorable physical properties such as melting points, viscosity, density, solubility and hydrophobicity have made RTILs ideal solvents for catalytic and synthetic reactions [97]. Excellent solubility of RTILs to dissolve a wide range of biomass matrices have them actively applied for extraction and separation of bioactive compounds [98]. High tailorability, good water solubility and biodegradability allow ionic liquids to gain penetration to ecological system for the purpose of drug delivery, drug synthesis, biomedical analysis in pharmaceutics and medicine [99]. The negligible volatility, high thermal stability, good electric conductivity and large electric window have enabled RTILs to serve not only as valuable electrolytes but also as precursors for carbon material electrodes [100]. Nonflammability, high thermal stability and oil-solubility satisfy the requirements for use as lubricants and lubricant additives [101]. Figure 6 gives structures of common cations and anions for ionic liquids.

For gas sensing purpose, ILs present great value specifically in its tunable structure and high adsorption for VOCs [96,102,103]. Liang and coworkers were first to report ionic liquid on QCM for VOCs [104]. The successful detection of VOCs molecules via modification of functional groups at cations and anions displayed the tailorability of ionic liquid [104]. Later, Jin and coworkers was able to obtain promising results through employment of an IL-based sensor array on QCM [105]. Flammable organic molecules (ethanol, benzene, heptane and dichloromethane) showed linear response with increase of concentration at both room and high temperatures, though a deviation occurred at high concentration for dichloromethane due to exceed of its own vapor pressure. Statistical analysis further showed that IL-based array can be promising through providing unique response patterns for each vapor [105]. In addition, further modification of ILs and incorporation with other materials (e.g., ethyl cellulose matrix, electrospun nanofiber, or carbon nanotubes) have led to successful sensing of CO_2_ and SO_2_ [106,107,108,109].

## 4. Ionic Liquid on QCM for Chemoselective Gas Sensing

**Figure 7:**
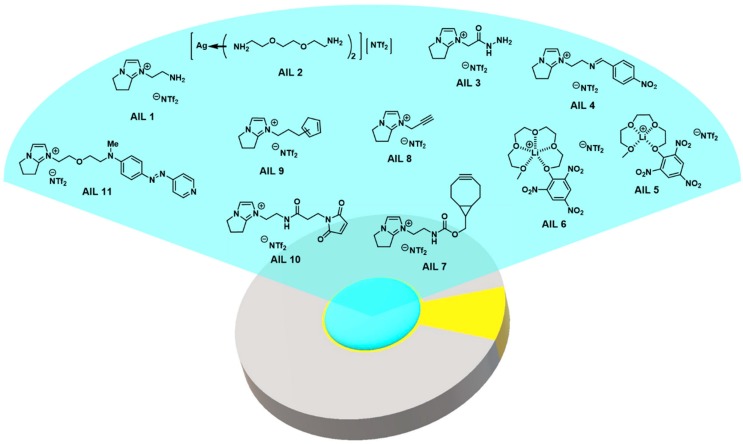
AILs are thin-coated on QCM electrodes for quantitative gas analysis. Here provides the AILs developed by Chu and coworkers for chemical reaction-based gas sensing.

Since 2010, we have put in efforts to the development of a series of affinity ionic liquids (AILs) targeted specifically for chemoselective gas sensing [110,111,112,113,114,115,116,117]. Albeit the bulk reports from others in the past have been successful in detecting gas molecules via physical adsorption, the challenge for those methods remains for better specificity to discriminate structurally similar gas molecules from others. Chemoselective gas analysis, on the contrary, can provide a simpler solution through inducing reaction-based selection via chemical reactivity of the compounds. In addition, we have been able to use this chemoselectivity as the advantage in successfully isolating target analytes from common VOCs interference (e.g., water, methanol, acetone, ethyl acetate, hexane, acetonitrile) at room temperature. Figure 7 and Table 1 provide the structures of AILs developed in our laboratory and mechanisms employed for chemoselectivity to target gas molecules.

### 4.1. Chemoselective Sensing of Aldehyde and Ketone Gases

Negligible vapor pressure and nonflammability have made room-temperature ionic liquids ideal materials to thin-coat on mass sensing transducer, QCM. The negligible vapor pressure ensures that ionic liquid does not dry out and is free of leakage during setups and experiments, while the nonflammable property allows for easy deposition of ionic liquid on QCM by dilution in methanol, which can be readily removed through short baking in oven.

**Figure 8:**
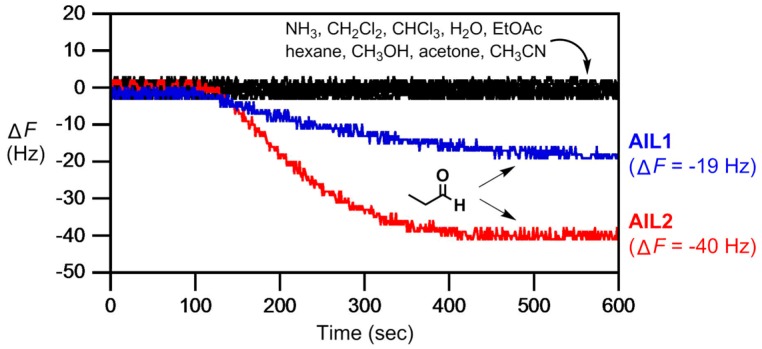
Chemoselective detection of common VOCs (e.g., ammonia, dichloromethane, chloroform, water, ethyl acetate, hexane, methanol, acetone, acetonitrile) and propionaldehyde gases at 100 ppb by a multi-channeled QCM thin-coated with **AIL1** and **AIL2** (33 nmol each, 200–300 nm thickness). Carrier gas (N_2_) had a flow rate of 3 mL/min and analyte injection was made at 100 s. A larger frequency change for propionaldehyde was observed for **AIL2**.

In 2010, we first reported **AIL1** on QCM for detection of gaseous aldehydes and ketones through the formation of a Schiff base by the imination of the amine group in **AIL1** [110]. Further quantitative studies were carried out to test selectivity between two model gases of identical molecular weight, butyraldehyde and 2-butanone, that is, to differentiate aldehyde from ketone. In coherent with its reactivity towards aldehyde, butyraldehyde was able to response better with higher sensitivity of detection. Furthermore, metal containing ionic liquid **AIL2** was also employed for gas analysis due to high affinity for alkylamine that resulted in easy preparation. Although **AIL2** has significant drawbacks in lesser stability under light; it exhibited a better response for propionaldehyde as evidenced in Figure 8 [111].

**Figure 9:**
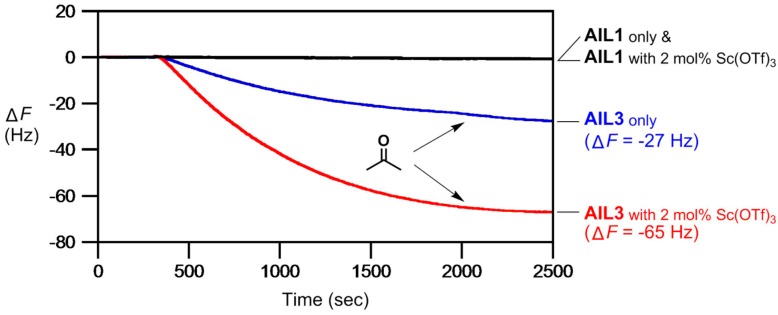
An ultrasensitive, 2 mol% Sc(OTf)_3_-catalyzed detection of acetone gas (58 ppb) by 9 MHz QCM thin-coated with **AIL3** (3.3 nL each, 300 nm thickness). **AIL1** was used here as the control ionic liquid. Gaseous acetone sample was injected at 300 s.

**Figure 10:**
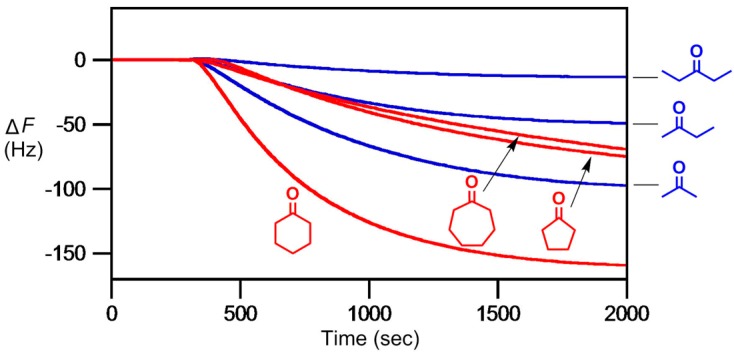
Chemoselective detection of acyclic and cyclic ketone gases (76 ppb each) on display by a 9 MHz QCM thin-coated with **AIL3** (3.3 nL, 300 nm thickness) with 2 mol% Sc(OTf)_3_. Ketone gas samples were injected at 300 s. Frequency drops for 3-pentanone, 2-butanone, acetone, cyclopentanone, cyclohexanone and cycloheptanone were 13, 49, 97, 75, 159 and 69 Hz, respectively.

In 2013, we went further to study chemoselective detection of gaseous ketones by using **AIL3** [112]. This is achieved by the formation of stable hydrazone adduct and a sensitive detection for low concentration of acetone (98 ppb) could be observed. Moreover, Lewis acid species were introduced to facilitate better hydrazone formation, with 2 mol% Sc(OTf)_3_ found to provide the most enhancement in detection sensitivity (Figure 9). For cyclic ketones, cyclopentanone, cyclohexanone (a signature compound emanating from C-4 explosive) and cycloheptanone were tested. Cyclohexanone displayed the largest response, in part of a greater reactivity with **AIL3** due to its sterically-free chair conformation (Figure 10) [112].

### 4.2. Chemoselective Sensing of Amine Gases

**Figure 11:**
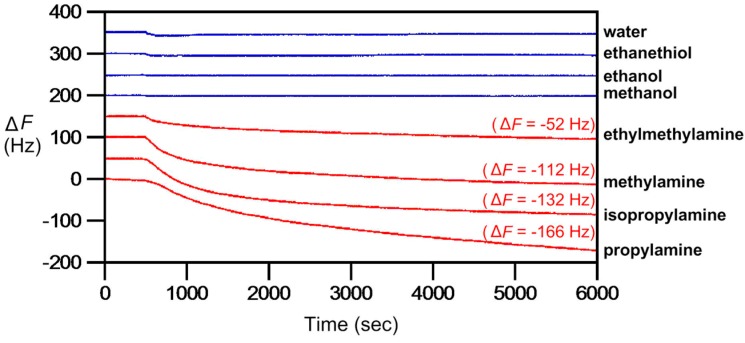
Chemoselective detection of water, ethanethiol, ethanol, methanol, ethylmethylamine, methylamine, isopropylamine and propylamine gases (100 ppb each) by 9 MHz QCM thin-coated with **AIL6**. The carrier gas had a flow rate of 3 mL/min and the analytes were injected at 500 s. The sensorgrams above propylamine were purposely shifted vertically (50 Hz in between) for clarity.

Modification of ionic liquids was made to demonstrate that, as an important part of VOCs as well as bacterial volatiles, amine gases could be readily captured and detected by **AIL4**, **5** and **6**. The working mechanism for **AIL4** is the transimination reaction. Good selectivity with high sensitivity results were obtained using propylamine as a model amine gas with the addition of 1 mol% Sc(OTf)_3_ to serve as a Lewis acid catalyst [110]. In 2017, Li and Chu reported the use of **AIL5** and **AIL6** for sensitive detection of amine gases by means of nucleophilic aromatic addition reaction [113]. Briefly, upon the introduction of amine gases, **AIL5** and **AIL6** form the Meisenheimer complexes with amine molecules at the electron deficient 2,4,6-trinitrophenyl (TNP) group in ionic liquids. Here the AILs served not only as amine-specific reacting agents but also for the purpose of accelerating the reactions through the TNP-containing arene, which serves as a super electrophile, resulting in the stabilization of the Meisenheimer complexes formed. QCM results demonstrated that the sensitivity of detection for **AIL5** and **AIL6** with propylamine gas at frequency drop of Δ*F* = −10 Hz was determined to be 8.0 and 5.4 ppb, respectively. Furthermore, steric hindrance that played into the reactivity was also observed, with the primary amines responding with larger Δ*F* values in respect to the more sterically hindered secondary amines (Figure 11). In addition to the mass sensing provided by QCM analysis, the Meisenheimer adduct, owing to the significant changes in electronic conjugation upon nucleophilic addition by amines at the aromatic ring carbon located at TNP, was able to display an orange red color visible by the naked eye. As reported, the instantaneous response of color change in **AIL6** upon exposure to amine gases when loaded on paper gives it potential for future applications as invisible ink or portable amine detector [113].

### 4.3. Chemoselective Sensing of Azide Gases

In 2014, we demonstrated the sensitive detection of organic azide gases through strain-promoted click reactions by **AIL7** on QCM. From the structure of **AIL7**, the strained triple bond on the cyclooctyne ring was able to display a stronger affinity for organic azide gases. Furthermore, a collection of azide gases were tested: propyl azide, pentyl azide, butyl azide, allyl azide and phenyl azide. **AIL7** exhibited great reactivities to these gases and, at Δ*F* = −10 Hz, sensitivity of detection for benzyl azide and butyl azide was 5 ppb and 35 ppb, respectively. Furthermore, in line with the activation energy study of azides, an order of frequency change on QCM was obtained: benzyl azide > phenyl azide > allyl azide. On the other hand, **AIL8**, as the control group was totally inert to the azide gases used. Not surprisingly, **AIL7** can also readily undergo the Diels-Alder [4 + 2] cycloaddition reaction with the cyclopentadiene gas [114].

### 4.4. Chemoselective Sensing of Alkene Gases

**Figure 12:**
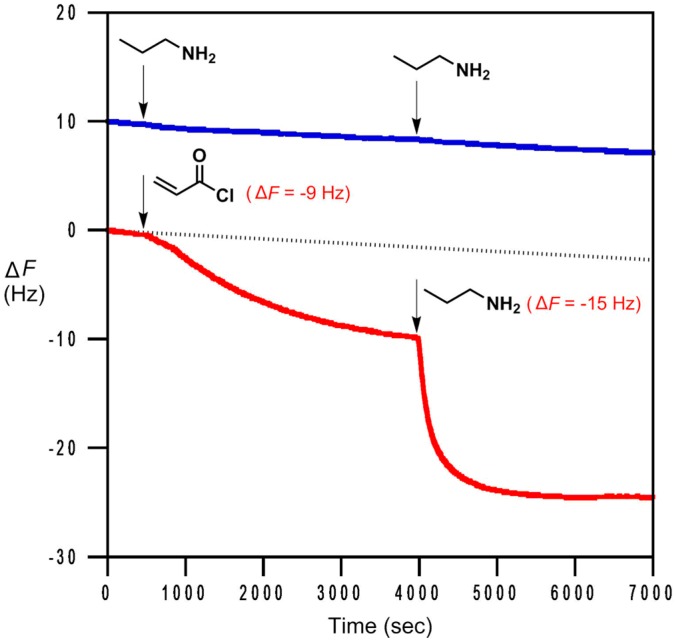
Detection of acryloyl chloride and propylamine (503 ppb each) was achieved by **AIL9**. Acryloyl chloride was injected at 500 s while propylamine was at 4000 s. The QCM sensorgrams for two injections of propylamine gas purposely shifted vertically by 10 Hz for clarity [112].

Later on, we incorporated a cyclopentadiene group in **AIL9** and a maleimide dienophile group in **AIL10** for QCM studies of its fast Diels-Alder [4 + 2] cycloaddition reactions with alkene and diene gases, respectively [115]. Sensing by **AIL9** was tested on five alkene gas molecules (1-pentene, cyclopentene, methyl acrylate, acrolein, acryloyl chloride), with acryloyl chloride producing the largest response in frequency drop. In addition, right after the first [4 + 2] cycloaddition reaction to afford the Diels-Alder adduct, the dual functionalized acryloyl chloride could be continued for a second consecutive reaction with an amine gas, a very fast Schotten-Baumann acylation, as demonstrated in Figure 12. Further on, **AIL10** was synthesized for effective diene gas analysis, also, based upon the Diels-Alder reaction. As expected, the addition of 5 mol% Sc(OTf)_3_ Lewis acid further promoted the Diels-Alder reaction and a linear QCM frequency response with increase of concentration of cyclopentadiene was obtained. A sensitivity of detection of 1.5 ppb at Δ*F* = −1 Hz was determined [115]. Next, among three other dienes (2,3-dimethyl-1,3-butadiene, cyclohexadiene and isoprene) investigated, **AIL10** was also able to demonstrate high specificity to cyclopentadiene gas due to the fact that only it adopts a coplanar s-*cis* conformation [115].

### 4.5. Chemoselective Sensing of Chemical Warfare Agent Mimics

**Figure 13:**
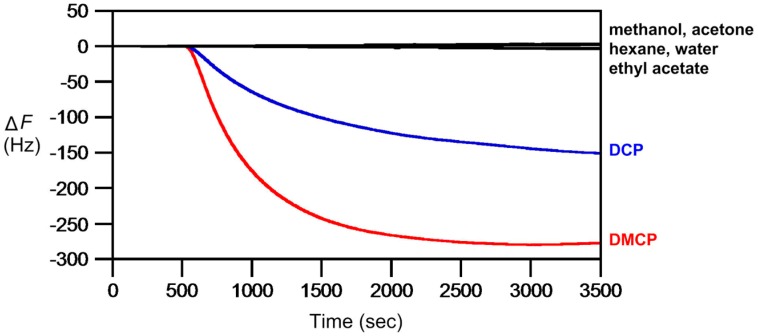
Chemoselective detection of common VOCs (water, ethyl acetate, hexane, methanol, acetone), DMCP and DCP at 526 ppb each by QCM thin-coated with **AIL11** (2.2 nL each, 200 nm thickness). Carrier gas (N_2_) had a flow rate of 3 mL/min and analyte injection was made at 500 s.

In 2018, to counter with gaseous chemical warfare agents (CWAs), **AIL11** were designed and synthesized specifically for vapor detection of nerve agents (G-agents) mimics, diethyl chlorophosphate (DCP) and dimethyl chlorophosphate (DMCP), in our laboratory [116]. The mechanism of reaction involved the highly reactive azopyridine on **AIL11,** which serves as a nucleophile to undergo substitution reaction. The resulting frequency changes, along with the color change in azobenzene chromophore, were analyzed for quantitative measurements. The sensitivity of detection of **AIL11** for DCP was determined to be 20 ppb at Δ*F* = −5 Hz. **AIL11** displays chemoselectivity to decipher DCP and its analogous, more volatile dimethyl chlorophosphate DMCP, from common VOCs (Figure 13). However, in retrospect to its successful detection of DCP and DMCP vapor, **AIL11** was found to be not reactive enough at ambient temperature to detect the vapor of 2-chloroethyl ethyl sulfide (CEES), a blister sulfur mustard (HD) mimic, due to the poor electrophilicity of CEES. Further studies are needed so that the appropriate modification can be made for its trace analysis.

## 5. Conclusions and Outlook

Real-time analysis of gaseous molecules remains a challenging task even with current state-of-the-art analytical tools. Broadly speaking, methods for gas detection can be separated into two parts. Spectroscopic and spectrometric methods are powerful tools to provide high sensitivity in detection, yet the shortcomings of their large sizes in instruments, immobility and relatively expensive cost make them unsuitable for immediate analysis [118]. On the other hand, inspired by the mammalian olfactory system, various concepts of electronic nose have been employed and developed. These conductivity and piezoelectric methods involved the collection of highly selective sensors to comprise an array for analysis of gaseous molecules via pattern recognition or any other classification algorithm. However, this seem-to-be ideal solution has yet to be achieved due to the fact that compounds of similar structures can be indistinguishable for most sensors and other limiting factors such as high operating temperatures for MOSs make them less straightforward for use.

AIL-on-QCM for chemoselective gas analysis has the potential to provide an alternative sensing system. Though subject to interference, QCM remains to be an electrochemical device that can provide a highly sensitive platform. The incorporation of AILs embedded with the unique chemical properties on QCM quartz electrodes make it a promising tool for array sensing of gas mixtures. Also to be noted are the further studies needed to address factors that may affect changes in the QCM resonant frequency, such as temperature and humidity. In addition, better engineering in functional groups is needed to assure competitive reactions with ionic liquids will not occur. Albeit idealization to mimic that of a dog’s nose is still beyond reach, the envision of a collection of AILs and perhaps a hybrid gas sensing system to complement each other may regard as a big step forward. After all, at the moment, not one method is satisfactory for all demands [117].

## Figures and Tables

**Table 1:** A brief description of the working principles AILs employed, the targeted analytes and the sensitivity of detection.

Ionic Liquid	Target Gas Species	Sensing Mechanism	Sensitivity of Detection	Literature
**AIL1**	aldehydes; ketones	imination	Δ*F* = −1.0 Hz: 4.5 ppb ^1^; 148 ppb ^2^	[110]
**AIL2**	aldehydes	imination	Δ*F* = −2.0 Hz: 4.6 ppb ^3^	[111]
**AIL3**	acyclic and cyclic ketones	hydrazone adduct formation	Δ*F* = −1.0 Hz: 0.6 ppb ^4^	[112]
**AIL4**	amines	transamination	Δ*F* = −1.0 Hz: 2.5 ppb ^5^	[110]
**AIL5**	amines	nucleophilic aromatic addition	Δ*F* = 10 Hz: 8.0 ppb ^6^	[113]
**AIL6**	amines	nucleophilic aromatic addition	Δ*F* = 10 Hz: 5.4 ppb ^7^	[113]
**AIL7**	azides	Huisgen 1,3-dipolar [3 + 2] cycloaddition	Δ*F* = 10 Hz: 5 ppb ^8^; 35 ppb ^9^	[114]
**AIL8**	control group	inert	inert	[114]
**AIL9**	dienes	Diels-Alder [4 + 2] cycloaddition	N/A	[115]
**AIL10**	dienes	Diels-Alder [4 + 2] cycloaddition	Δ*F* = −1 Hz: 1.5 ppb ^10^	[115]
**AIL11**	CWA mimics	nucleophilic substitution	Δ*F* = 5 Hz: 20 ppb ^11^	[116]

Sensitivity of detection with respect to ^1^ butyraldehyde; ^2^ 2-butanone; ^3^ propionaldeyhyde; ^4^ cyclohexanone with 2 mol% Sc(OTf)_3_; ^5^ propylamine with 1 mol% Sc(OTf)_3_; ^6,7^ propylamine; ^8^ benzyl azide; ^9^ butyl azide; ^10^ cyclopentadiene; ^11^ diethyl chlorophosphate.
